# Prevalence of chronic pelvic pain and primary dysmenorrhea in women of reproductive age in Ecuador

**DOI:** 10.1186/s12905-022-01948-y

**Published:** 2022-09-02

**Authors:** Carmen Yolanda de Las Mercedes Villa Rosero, Suleimy Cristina Mazin, Antonio Alberto Nogueira, José Antonio Vargas-Costales, Julio Cesar Rosa-e-Silva, Francisco José Candido-dos-Reis, Omero Benedicto Poli-Neto

**Affiliations:** 1 Department of Obstetrics and Gynecology, Universidad Central del Ecuador, Quito, Ecuador; 2 Laboratory for Translational Data Science, Department of Obstetrics and Gynecology, Ribeirão Preto Medical School of the University of São Paulo USP, 3900, Bandeirantes Avenue. Monte Alegre, Ribeirão Preto, SP 14.049-900 Brazil; 3 Department of Pharmacology, School of Medicine, Faculty of Medical Sciences, Central University of Ecuador, Quito, Ecuador

**Keywords:** Chronic pelvic pain, Primary dysmenorrhoea, Prevalence, Associated factors, Ecuador

## Abstract

**Background:**

Chronic pelvic pain (CPP) and primary dysmenorrhoea are debilitating conditions that can impair the quality of life of affected women. These conditions are frequently neglected, delaying proper diagnosis and healthcare provision. This study aimed to estimate the prevalence of CPP and primary dysmenorrhoea in Ecuador and identify potential variables associated with their occurrence.

**Methods:**

We conducted a cross-sectional survey in an urban neighbourhood of Quito, the capital of Ecuador. A total of 2397 participants of 14–49 years of age were included. The data were collected through questionnaires administered by trained interviewers.The crude and adjusted prevalence ratios were calculated using a log-binomial regression model. The correlation between pain intensity catastrophising of symptoms were statistically analysed.

**Results:**

The prevalence of CPP and primary dysmenorrhoea was 9.8% and 8.9%, respectively. Irritative urinary symptoms, primary dysmenorrhoea, and underlying mental disorders were associated with CPP, while smoking, irritable bowel syndrome, sleep disturbance, dyspareunia, and mental disorders were associated with primary dysmenorrhoea.

**Conclusions:**

The prevalence of CPP and primary dysmenorrhoea in Ecuador was similar to that in other Latin American countries. Primary dysmenorrhoea is a risk factor of CPP, and less than a quarter of women are undergoing treatment for the condition. Our findings reinforce the importance of healthcare interventions in anticipating the diagnosis of these conditions in women of reproductive age.

**Supplementary Information:**

The online version contains supplementary material available at 10.1186/s12905-022-01948-y.

## Background

Chronic pelvic pain (CPP) is a common condition that affects women at different stages of life, but most often occurs during the reproductive years [1]. As a role, it is defined as cyclical or non-cyclical pain of at least 3–6 months’ duration that occurs in lower abdominal region, pelvis, or the female organs of women, usually associated with negative cognitive, behavioral, sexual and emotional consequences. Despite recent efforts, there is still no consensus on a precise definition [2]. Typically, chronic cyclical pelvic pain is considered pain that occurs in association with the menstrual cycle, including dysmenorrhoea, but not only. It also incorporates pelvic pain that occurs in a cyclic pattern and not related to the menstrual cycle, such as those that occur at the time of intercourse and ovulation (Mittelschmerz pain) or are associated with numerous pathologies [3]. Primary dysmenorrhoea is the most frequent cause of cyclical CPP. It is defined as painful menstrual cycle with a cramping sensation in the lower abdomen immediately before or during the menstruation period in the absence of any pelvic pathology and commonly accompanied by other symptoms, such as sweating, headache, nausea, vomiting, diarrhoea, and tremulousness [4]. Typically it is presented after menarche or shortly after it (6 to 12 months) and is reported by up to 75% of women, being severe in up to 15% of them [5–8].

Non-cyclical CPP does not maintain a relationship with the cycle. Its global prevalence varies from 2%–27% and is approximately 4% in developed countries [9, 10]. At least 20% of these women do not have their condition properly investigated, up to 60% remain undiagnosed, and many experience symptoms for long periods [11, 12]. An overlap of gynaecological and non-gynaecological conditions can be diagnosed in up to 60% of women [13], and in approximately one-third of the patients, no specific pelvic disease is identified [14]. CPP is often associated with mood disorders [15], and 60%–80% of patients are diagnosed with somatoform disorder according to the International Classification of Diseases-10 criteria [16]. CPP accounts for 10%–20% of gynaecological consultations, 20% of hysterectomies, and 40% of gynaecological laparoscopies [17]. In addition, the disease has a negative impact on women's quality of life [18], contributes to high catastrophising scores [19], leads to social isolation [20], has a negative impact on performing work and daily activities [21], contributes to the frequent use of health services [22], and has a significant economic impact on the lives of women and the community as a whole [23, 24]. All these factors make the condition a serious public health problem that is still underestimated.

From a pathophysiological point of view, the condition is probably not a consequence of a specific disease, but a complex interaction of diverse factors, including life history and sociocultural factors. Several association factors have been identified in women with non-cyclic CPP and/or dysmenorrhoea, including age < 30 years, low body mass index, smoking, menarche before 12 years of age, long menstrual cycles, prolonged menstrual flow, nulliparity, premenstrual syndrome, infertility, pelvic inflammatory disease, sexual abuse, childhood violence, psychological symptoms, alcohol and drug abuse, abortions, endometriosis, and previous caesarean section [25–27]. The interaction between these factors and other correlates, such as high body mass index, catastrophising, and pelvic floor tenderness, has also been identified through machine learning tools [28]. Although a direct causal relationship between these associations and CPP cannot be inferred, there is an evident interaction between the gynaecological, urinary, gastrointestinal, musculoskeletal, neuroendocrine, and psychological systems.

Currently, there is a scarcity of studies reporting the prevalence of CPP and associated factors, making it difficult to design effective global public health policies to mitigate this problem. Mapping the occurrence of CPP and identifying associated factors in different countries and regions can contribute to a better understanding of the nuances of the disease, and consequently, favour measures to improve women’s health. Our objective was to estimate the prevalence of CPP, particularly non-cyclical pelvic pain (not occurring in association with menstruation or in a temporal pattern) and primary dysmenorrhoea (painful menstrual cramps presented since menarche), among women in the urban community of Quito, the capital of Ecuador, and to identify potential variables associated with their occurrence.

## Methods

### Study design

A cross-sectional community-based survey was conducted that included 2397 women of 14–49 years of age, recruited between August 2017 and July 2018 in Quito, Ecuador. This study was approved by the relevant university ethics committee. All participants or their legal representatives provided written informed consent. We followed the ethical standards for the regulation of research in humans in accordance with the Declaration of Helsinki.

Women of reproductive age between 14 and 49 years on the date of the interview, residing in the urban parishes of the Metropolitan District of Quito were eligible for inclusion. We excluded women who had been pregnant during the 12 months prior to the interview or those with a cognitive deficiency or altered mental state that prevented obtaining informed consent, understanding the questionnaire, or completing the interview.

### Interviewer training and data management

Initially, two physicians supervising the interviewers underwent face-to-face training at the pelvic pain clinic, a specialized multidisciplinary public service and national reference with more than 25 years of experience in caring for these women. Subsequently, “in locu” interviewers were selected and trained. All interviewers had knowledge in the health area, but were not directly linked to any public health care program. The questionnaire (Additional file 1) was pre-tested on 50 women randomly selected from the urban parishes in the Metropolitan District of Quito. All women who were diagnosed with CPP were referred to the public health network for evaluation of their condition. Data were collected and managed using the REDCap electronic data capture tools hosted at [https://redcap.fmrp.usp.br/] [29]. REDCap is a secure, web-based software platform designed to support data capture for research studies, providing (1) An intuitive interface for validated data capture, (2) Audit trails for tracking data manipulation and export procedures, (3) Automated export procedures for seamless data downloads to common statistical packages, and (4) Procedures for data integration and interoperability with external sources.

### Research location

The study was conducted in an urban area of the Metropolitan District of Quito, or the canton of Quito. The city is located in the province of Pichincha in northern Ecuador, in the inter-Andean region, in the eastern part of the Andes, and is divided into units of lower administrative political hierarchy, the parishes. In Quito, it is estimated that there are more than 830,000 women of reproductive age (between 14 and 49 years of age), accounting for approximately 57.3% of the population.

### Data source and measurement methods

**Fig. 1:**
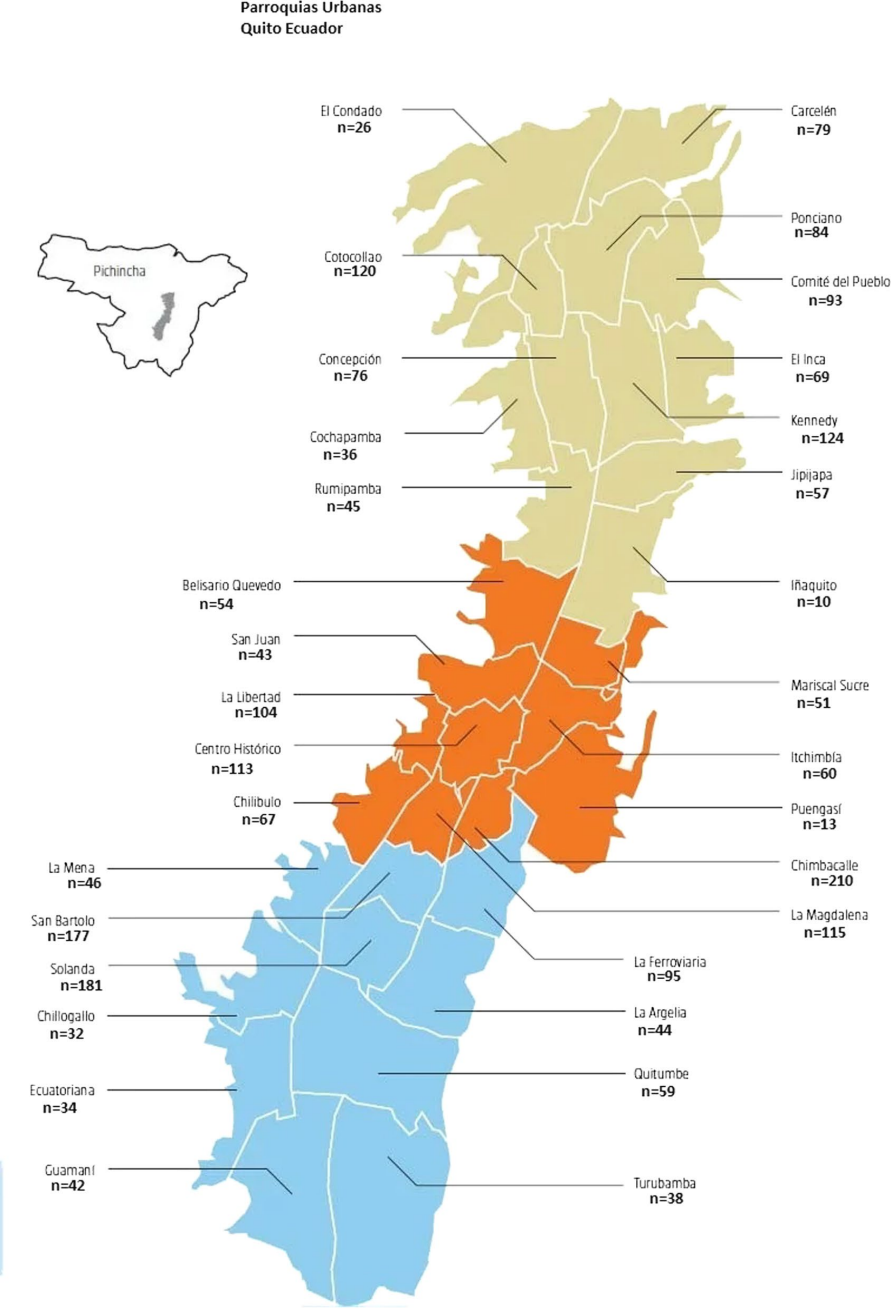
Urban parishes "parroquias" in Quito Ecuador and number of interviews carried out in each one

Information was obtained through interviews conducted at home in a confidential environment at a time chosen by the participant. A proportional stratified probabilistic sample was obtained, considering the population density per hectare in each parish (Fig. 1). The addresses in each region were selected by systematic sampling, in which residences were selected at regular intervals considering the number of homes estimated in the parish and the estimated number of women living in that area. Only one woman in each household was interviewed. In case of more than one woman being interested in participating by residence, the participant was randomly selected through the roll of a die (the one with the highest value would be chosen). The questionnaire chosen as the data collection instrument was provided to and completed by the interviewee herself or with the help of the interviewer, guaranteeing confidentiality and avoiding the effect of excessive interference from the researcher.

### Sample size

The sample size was calculated according to the study design to respond to the main objective of the study, considering an infinite population. For this we used the expression $n=\frac{{z}_{\frac{\alpha }{2}}^{2}\left(1-P\right)}{{\epsilon }^{2}P}$, where is the sample size, ${z}_{\frac{\alpha }{2}}^{2}$ is the statistic corresponding to level of confidence, *P* is the expected prevalence, and $\epsilon$ is the relative error (the difference between the observed and expected prevalence multiplied by 100 and divided by the expected) [30]. We performed a simulation in which the prevalence and relative error were varied to estimate the sample size. We considered a scenario with a prevalence of 4% for both outcomes, a confidence coefficient of 95%, and a relative error of 20% as the most appropriate, and subsequently decided to survey at least 2,305 women.

### Biases and minimization methods

The interviews were conducted predominantly on Saturday afternoons\because it was the most suitable period identified when all residents of the household were expected to be at home. All the women in the house at the time of the interviewers visit were screened for participation. In the event that more than one potential participant met the eligibility criteria in a single household, we performed a simple random selection, so that only one woman from each household was included. Children younger than 18 years participated if their legal representatives were found to authorise it.

Approximately 10% of the women in each parish were re-interviewed to check the reliability of the information provided. Any disagreement was reaffirmed with the participant and the information was considered ratified in the last interview. The data analysis was conducted by one of the researchers who did not have access to the interviews and by a statistician without prior knowledge of the clinical or characterisation data of the participants.

### Variables

To define CPP, we considered the reVITALize proposal, an initiative led by the American College of Obstetricians and Gynecologists, that aims to standardise the terminology in gynaecology and obstetrics [31]. Therefore, we defined CPP as “pain symptoms perceived to originate from pelvic organs or structures typically lasting more than six months. It is often associated with negative cognitive, behavioural, sexual, and emotional consequences and symptoms suggestive of lower urinary tract, sexual, bowel, pelvic floor, myofascial, or gynaecological dysfunction”. The definition used was also in accordance with that proposed by the International Association for the Study of Pain [32]. To assess non-cyclical pelvic pain we asked the following question: “Have you ever experienced any persistent pelvic pain? Consider any type of pain in the lower part of your belly not occurring in concert with menstruation or in a temporal pattern. At this moment, you should not consider pain related to stomach flu, acute and infectious diarrhoea, food poisoning, acute trauma, sports, surgery, pregnancy or childbirth, intercourse, periods, menstrual cramps”. To assess primary dysmenorrhea we asked the following question: “Have you experienced recurrent, cramping pain during your periods since around your first period?”. Cramps perceived during, or shortly before and after, the menstrual period were considered, which characteristically began near menarche [4]. Pain from sexual intercourse is controversial but has been discussed as a component symptom of the conditions studied [13, 31]. Therefore, we assessed pain from sexual intercourse, but did not consider it part of CPP. We consider only moderate (intense pain that interrupts sexual intercourse) and severe dyspareunia (intense pain that prevents sexual intercourse).

We analysed non-cyclical pelvic pain and primary dysmenorrhoea separately as dependent variables and as a joint variable (non-cyclical pelvic pain plus primary dysmenorrhea). Pain intensity was measured using a visual analogue scale (VAS). The VAS consists of a 100-mm-long line at the left end of which is the phrase, “I don’t feel any pain,” and at the right end, “The pain I feel can’t be greater.” This scale is practical, fast, widely accepted, valid, and reliable [33, 34]. Participants were considered to have significant non-cyclical pelvic pain when the VAS score was ≥ 30 mm with a frequency of at least one episode per week [35]. The intensity of dysmenorrhoea was also classified as mild, moderate, or severe according to the degree of systemic involvement, use of medications, and level of interference with work or daily activities. Thus, dysmenorrhoea was classified as mild (possible pelvic discomfort with no effect on daily activity with, need for occasional medication), moderate (pain that affects daily life, responds to the use of medications, and forces you to miss work or school), or severe (pain that lasts the entire menstrual period, with significant limitations in daily activities, frequent use of high dosage medications, forces you to miss work, and often accompanied by symptoms such as severe headaches, weakness, and vomiting). At the time of the analysis, we considered only moderate and severe dysmenorrhoea as significant risk factors of CPP.

The other variables analysed were social, gynaecological, and clinical factors. The social metrics included age, body mass index, marital status, level of education, income, physical activity, religion, smoking of cigarettes, alcohol use disorder (impaired ability to stop or control alcohol use despite adverse social, occupational, or health consequences) according to criteria of the Diagnostic and Statistical Manual-5 [36], illicit drug use, and whether they had previously been the victim of violence (physical or sexual). Gynaecological variables included the use of contraceptives, sexual activity, dyspareunia (genital pain that can be experienced before, during, or after intercourse) [37], dysmenorrhoea, abnormal menstrual pattern (periods that occur less than 21 days or more than 45 days apart, duration higher than 8 days, missing three or more periods in row, and menstrual flow that is much higher or lower than usual) [38], and parity. The clinical variables were heart disease, diabetes, respiratory disease, previous completed cancer treatment, diagnosis of a mental disorder, health conditions such as low back pain and headache/migraine, infra-umbilical surgery (including caesarean section), functional gastrointestinal disorders diagnosed by the Rome-III criteria [39] and the Bristol Stool Form Scale [40, 41], urinary symptoms (pain, urgency, and increased frequency), and sleep disturbances. Additionally, the presence of mental disorders was determined using the self-reporting questionnaire 20 (SRQ-20) developed by the World Health Organization [42]. The SRQ-20 is used as a screening or case-finding instrument for identification of important psychological symptoms commonly in primary care settings. The instrument has been shown to be reproducible and consistent in various countries and communities. It has been used in various countries of Latin America [43–45] including Ecuador [46] as observed in previous studies, we used a cut-off > 7 to consider a positive screening for mental disorder. Pain catastrophizing was assessed using the Spanish version of Pain Catastrophising Scale, which has shown excellent reliability [47].

### Statistical methods

All statistical analyses were performed using SAS version 9.4 (SAS Institute Inc., Cary, NC, USA, 2011). Initially, an exploratory data analysis was performed, considering the measures of central position (mean and median) and dispersion (standard deviation). For qualitative variables, absolute and relative frequencies were estimated. We used Student's t-test to compare the quantitative variables of interest, and Pearson correlation test was used to analyse the correlation between pain intensity and catastrophising of symptoms. To verify whether there was an association between the exploratory variables and their respective results, crude and adjusted prevalence ratios were calculated using log-binomial regression model. This model is a generalised linear binomial model with a logarithmic link function [48]. These models estimated the adjusted prevalence ratio followed by the 95% confidence interval. For each result, two models were built: one considering all the variables of interest, and another including only the variables that showed some statistical evidence of association on univariate analysis, considering all of them, even those significant for some outcomes only. However, in some situations we had restricted maximum likelihood limitations and the model did not fit. For this reason, there is a small divergence in the variables used to adjust the final models according to the outcome considered.Akaike’s information criteria, Bayesian information criteria, and log-likelihood criteria were considered for the choice of models.

## Results

We interviewed 2397 women. Thirty-three women residing in twenty residences chose not to participate in the research and did not respond to the questionnaires.

### Non-cyclical CPP

We identified 236 participants with non-cyclical CPP (9.8%). Among them, the mean pain intensity in the last three months was 60.3 ± 19.7 mm, and the pain catastrophising score was 11.1 ± 12.0 of possible 52 points. There was no correlation between pain intensity and the catastrophising score (r = 0.26, *p* = 0.066).

Table 1 shows the results of the questionnaire, divided into a control group and those with non-cyclical CPP. Table 2A presents the adjusted prevalence ratios, 95% confidence intervals and p-values for factors associated with CPP. Irritative urinary symptoms, primary dysmenorrhoea, and mental disorders were factors independently associated with CPP.

**Table 1:** Distribution of the variables regarding non-cyclical pelvic pain as the outcome

	N (%)	Control	CPP	PR	CI 95%	
		(n = 2161)	(n = 236)		inf	sup
Age, y						
< 20	363 (15.1)	339 (15.7)	24 (10.2)	0.76	0.50	1.15
20–35	1455 (60.7)	1328 (61.4)	127 (53.8)	1.00	1.00	1.00
> 35	579 (24.2)	494 (22.9)	85 (36.0)	1.68	1.30	2.18
Body mass index, Kg·m^−2^						
< 25	1457 (60.8)	1339 (62.0)	118 (50.0)	1.00	1.00	1.00
25–30	731 (30.5)	649 (30.0)	82 (34.8)	1.38	1.06	1.81
> 30	209 (8.7)	173 (8.0)	36 (15.2)	2.13	1.51	3.00
Marital status						
Married/stable union	1486 (62.0)	1311 (60.7)	175 (74.2)	1.00	1.00	1.00
Single, widow	829 (34.6)	779 (36.1)	50 (21.2)	0.51	0.38	0.69
Divorced	80 (3.3)	69 (3.2)	11 (4.66)	1.17	0.66	2.06
Educational level						
Low (< 8 years)	266 (11.1)	236 (11.0)	30 (12.7)	1.10	0.76	1.58
Intermediate (8–12 years)	1515 (63.4)	1359 (63.1)	156 (66.1)	1.00	1.00	1.00
High (> 12 years)	609 (25.5)	559 (26.0)	50 (21.2)	0.80	0.59	1.08
Occupation						
Employee	1019 (42,69)	900 (41,84)	119 (50,42)	1.00	1.00	1.00
Housewife	301 (12,61)	264 (12,27)	37 (15,68)	1,05	0,74	1.49
In education	985 (41,27)	908 (42,21)	77 (32,63)	0,67	0,51	0.88
Unemployed	82 (3,44)	79 (3,67)	3 (1,27)	0,31	0,10	0.96
Remuneration	1,121 (46.8)	998 (46.2)	123 (52.1)	0.81	0.64	1.03
Physical activity	979 (40.9)	882 (40.8)	97 (41.1)	1.01	0.79	1.29
Religious practice	1,698 (70.9)	1521 (70.4)	177 (75.0)	1.23	0.93	1.63
Smoking	474 (19.8)	411 (19.0)	63 (26.7)	1.48	1.13	1.94
Alcohol use disorder	1358 (56.6)	1,193 (55.2)	165 (69.9)	1.78	1.36	2.32
Illicit drug use	131 (5.5)	108 (5.0)	23 (9.8)	1.87	1.26	2.77
Violence victim	565 (23.6)	479 (22.2)	86 (36.4)	1.86	1.45	2.38
Abdominal surgery	668 (27.9)	567 (26.3)	101 (42.8)	1.93	1.52	2.46
Contraceptive use	620 (25.9)	571 (26.4)	49 (20.8)	0.75	0.56	1.02
Abnormal menstruation	431 (18.0)	369 (17.1)	62 (26.3)	1.62	1.24	2.13
Primary dysmenorrhoea	213 (8.9)	169 (7.8)	44 (18.6)	2.35	1.75	3.16
Previous sexual intercourse	1909 (79.6)	1702 (78.8)	207 (87.7)	1.82	1.25	2.66
Parity						
0	1239 (51.7)	1152 (53.3)	87 (36.9)	1.00	1.00	1.00
1–2	747 (31.2)	660 (30.5)	87 (36.9)	1.66	1.25	2.20
3 +	411 (17.2)	349 (16.2)	62 (26.3)	2.15	1.58	2.92
Dyspareunia	210 (16.1)	170 (14.9)	40 (26.1)	1.83	1.32	2.54
Cardiovascular disease	142 (5.9)	121 (5.6)	21 (8.9)	1.55	1.02	2.35
Diabetes	33 (1.4)	27 (1.2)	6 (2.5)	1.87	0.90	3.89
Respiratory disease	176 (7.3)	152 (7.0)	24 (10.2)	1.43	0.96	2.12
Cancer	17 (0.7)	13 (0.6)	4 (1.7)	2.41	1.02	5.74
Psychiatric disorder	77 (3.2)	61 (2.8)	16 (6.8)	2.19	1.39	3.45
Migraine/chronic headache	552 (23.0)	471 (21.8)	81 (34.3)	1.75	1.36	2.25
Lombalgia	382 (15.9)	317 (14.7)	65 (27.5)	2.00	1.54	2.61
Irritative urinary symptoms	373 (15.6)	302 (14.0)	71 (30.1)	2.34	1.81	3.01
Constipation	482 (20.1)	456 (21.1)	26 (11.0)	0.49	0.33	0.73
Distension	937 (39.2)	793 (36.8)	144 (61.0)	2.43	1.89	3.11
IBS criteria	283 (11.8)	162 (7.5)	121 (51.3)	7.86	6.29	9.82
Bristol scale						
Types 1–2	675 (28.2)	609 (28.2)	66 (28.0)	1.00	0.76	1.31
Types 3–4–5	1650 (68.9)	1488 (68.9)	162 (68.6)	1.00	1.00	1.00
Types 6–7	71 (3.0)	63 (2.9)	8 (3.4)	1.15	0.59	2.24
Sleep disturbance	654 (27.3)	559 (25.9)	95 (40.2)	1.80	1.41	2.29
SRQ > = 8	425 (17.7)	349 (16.2)	76 (32.2)	2.20	1.71	2.84

*CI* confidence interval; *CPP* non-cyclical chronic pelvic pain; *PR* prevalence ratio; *IBS* irritable bowel syndrome; *SRQ* self report questionnaire (used for screening mental disorders)

**Table 2:** Estimates of the prevalence ratio adjusted by the log-binomial model, followed by confidence intervals and the p-value considering women with non cyclical pelvic pain, primary dysmenorrhoea, and with both jointly conditions

	PR adjusted	CI 95%		*p*-value
		inf	sup	
A-Non cyclical pelvic pain^1^				
Irritative urinary symptoms	1.5	1.10	2.08	0.011
Primary dysmenorrhoea	1.5	1.00	2.12	0.047
Screening for mental disorders	1.4	1.02	1.94	0.038
B-Primary dysmenorrhoea^2^				
IBS criteria	2.2	1.45	3.27	0.002
Smoking	1.9	1.32	2.76	0.001
Dyspareunia	1.6	1.03	2.30	0.036
Sleep disturbance	1.6	1.08	2.28	0.018
Screening for mental disorders	1.5	1.06	2.38	0.025
C-Joint pain^3^				
IBS criteria	8.2	4.36	15.61	< 0.001
Screening for mental disorders	2.3	1.20	4.38	0.002

*CI* confidence interval; *IBS* irritable bowel syndrome; *PR* prevalence ratio

^1^Additional variables used to adjust the model: age, occupation, smoking, alcohol use disorder, violence, abdominal surgery, contraceptive use, abnormal menstruation, dispareunia, parity, cardiovascular disease, cancer, psychiatric disorder, migraine/chronic headache, lombalgia, constipation, distension, irritable bowel syndrome, sleep disturbance

^2^Additional variables used to adjust the model: body mass index, illicit drug use, violence, abnormal menstruation, abdominal surgery, migraine/chronic headache, irritative urinary symptoms, distension

^3^Additional variables used to adjust the model: irritative urinary symptoms, violence victim, sleep disturbance

### Primary dysmenorrhoea

We identified 213 participants with primary dysmenorrhoea (8.9%), of which 44 had non-cyclical CPP (20.7%). The mean pain intensity in the last three months was 74.3 ± 22.6 mm, and the pain catastrophising score was 12.8 ± 14.1. There was no correlation between the two variables (r = 0.25, *p* = 0.062). Table 3 shows the results of the questionnaire, divided into a control group and those with primary dysmenorrhoea. Table 2B presents the adjusted prevalence ratios, 95% confidence intervals and respective *p*-values of factors associated with primary dysmenorrhoea. Smoking, irritable bowel syndrome (IBS), sleep disturbance, dyspareunia, and mental disorder were independently associated with primary dysmenorrhoea.

**Table 3:** Distribution of the variables regarding primary dysmenorrhoea as the outcome

	N (%)	Control	Dysmenorrhoea	PR	CI 95%	
		(n = 2,184)	(n = 213)		inf	sup
Age, y						
< 20	363 (15.1)	324 (14.8)	39 (18.3)	1.21	0.86	1.70
20–35	1455 (60.7)	1326 (60.7)	129 (60.6)	1.00	1.00	1.00
> 35	579 (24.2)	534 (24.4)	45 (21.1)	0.88	0.63	1.21
Body mass index, Kg·m^−2^						
< 25	1457 (60.8)	1339 (61.3)	118 (55.4)	1.00	1.00	1.00
25–30	731 (30.5)	663 (30.4)	68 (31.9)	1.15	0.86	1.53
> 30	209 (8.7)	182 (8.3)	27 (12.7)	1.60	1.08	2.36
Marital status						
Married/stable union	1486 (62.0)	1358 (62.2)	128 (60.1)	1.00	1.00	1.00
Single, widow	829 (34.6)	751 (34.4)	78 (36.6)	1.09	0.84	1.43
Divorced	80 (3.3)	73 (3.4)	7 (3.3)	1.02	0.49	2.10
Educational level						
Low (< 8 years)	266 (11.1)	247 (11.4)	19 (8.9)	0.75	0.47	1.19
Intermediate (8–12 years)	1515 (63.4)	1371 (63.0)	144 (67.6)	1.00	1.00	1.00
High (> 12 years)	609 (25.5)	559 (25.7)	50 (23.5)	0.86	0.64	1.18
Occupation						
Employee	1019 (42.7)	922 (42.4)	97 (45.5)	1.00	1.00	1.00
Housewife	301 (12.6)	275 (12.6)	26 (12.2)	0.91	0.60	1.37
In education	985 (41.3)	899 (41.4)	86 (40.4)	0.92	0.70	1.21
Unemployed	82 (3.4)	78 (3.6)	4 (1.9)	0.51	0.19	1.36
Remuneration	1121 (46.8)	1020 (46.8)	101 (47.4)	0.98	0.76	1.26
Physical activity	979 (40.9)	889 (40.7)	90 (42.2)	1.06	0.82	1.37
Religious practice	1698 (70.9)	1544(70.7)	154 (72.3)	1.07	0.80	1.43
Smoking	474 (19.8)	414 (19.0)	60 (28.2)	1.59	1.20	2.11
Alcohol use disorder	1358 (56.6)	1227 (56.2)	131 (61.5)	1.22	0.94	1.59
Illicit drug use	131 (5.5)	111 (5.1)	20 (9.4)	1.79	1.17	2.74
Violence victim	565 (23.6)	486 (22.2)	79 (37.1)	1.91	1.47	2.48
Abdominal surgery	668 (27.9)	592 (27.1)	76 (35.7)	1.44	1.11	1.88
Contraceptive use	620 (25.9)	567 (26.0)	53 (24.9)	0.95	0.71	1.28
Abnormal menstruation	431 (18.0)	381 (17.4)	50 (23.5)	1.40	1.04	1.89
Previous sexual intercourse	1909 (79.6)	1727 (79.1)	182 (85.4)	1.50	1.04	2.17
Parity						
0	1224 (51.1)	1114 (51.0)	110 (51.6)	1.00	1.00	1.00
1–2	752 (31.4)	675 (30.9)	77 (36.2)	1.08	0.73	1.60
3 +	411 (17.2)	372 (17.2)	49 (17.0)	1.35	0.88	2.07
Dyspareunia	210 (16.2)	170 (14.6)	40 (30.8)	2.30	1.63	3.24
Cardiovascular disease	142 (5.9)	130 (6.0)	12 (5.6)	0.95	0.54	1.66
Diabetes	33 (1.4)	27 (1.2)	6 (2.8)	2.08	1.00	4.33
Respiratory disease	176 (7.3)	154 (7.0)	22 (10.3)	1.45	0.96	2.20
Cancer	17 (0.7)	17 (0.8)	0 (0.0)	–	–	–
Psychiatric disorder	77 (3.2)	66 (3.0)	11 (5.2)	1.64	0.94	2.88
Migraine/chronic headache	552 (23.0)	479 (21.9)	73 (34.3)	1.51	1.06	2.16
Lombalgia	382 (15.9)	339 (15.5)	43 (20.2)	1.13	0.70	1.82
Irritative urinary symptoms	373 (15.6)	319 (14.6)	54 (25.4)	1.84	1.38	2.46
Constipation	482 (20.1)	443 (20.3)	39 (18.3)	0.89	0.64	1.24
Distension	937 (39.1)	824 (37.7)	113 (53.0)	1.77	1.37	2.29
IBS criteria	283 (11.8)	220 (10.1)	63 (29.6)	3.14	2.40	4.10
Bristol scale						
Types 1–2	675 (28.2)	612 (28.0)	63 (29.6)	1.05	0.80	1.40
Types 3–4–5	1650 (68.9)	1503 (68.9)	146 (68.5)	1.00	1.00	1.00
Types 6–7	71 (3.0)	67 (3.1)	4 (1.9)	0.64	0.24	1.67
Sleep disturbance	654 (27.3)	529 (24.2)	125 (58.7)	2.61	1.86	3.65
SRQ > = 8	425 (17.7)	352 (16.1)	73 (34.3)	2.30	1.63	3.24

*CI* confidence interval; *PR* prevalence ratio; *IBS* irritable bowel syndrome; *SRQ* self report questionnaire (used for screening mental disorders)

#### Non-cyclical CPP plus primary dysmenorrhoea

We identified 44 participants with non-cyclical CPP and primary dysmenorrhoea (1.8%). Among them, the mean pain intensity in the last three months was 80.9 ± 17.8 mm, and the pain catastrophising score was 16.9 ± 13.8 of possible 52 points. There was no correlation between pain intensity and the catastrophising score (r = 0.07, *p* = 0.640).

Table 4 shows the results of the questionnaire, divided into a control group and those with non-cyclical CPP and primary dysmenorrhoea. Table 2C presents the adjusted prevalence ratios, 95% confidence intervals and *p*-values for factors associated with joint conditions. IBS and mental disorders were independently associated with these joint conditions.

**Table 4:** Distribution of the variables regarding the joint variables non-cyclical pelvic pain and primary dysmenorrhoea (joint pain) as the outcome

	N (%)	Control	Joint pain	PR	CI 95%	
		(n = 2353)	(n = 44)		inf	sup
Age, y						
< 20	363 (15.1)	357 (15.2)	6 (13.6)	1.05	0.43	2.55
20–35	1455 (60.7)	1432 (60.9)	23 (52.3)	Ref	Ref	Ref
> 35	579 (24.2)	564 (24.0)	15 (34.1)	1.64	0.86	3.12
Body mass index, Kg·m^−2^						
< 25	1457 (60.8)	1435 (61.0)	22 (50)	Ref	Ref	Ref
25–30	731 (30.5)	715 (30.4)	16 (36.4)	1.45	0.77	2.74
> 30	209 (8.7)	203 (8.6)	6 (13.6)	1.90	0.78	4.63
Marital status						
Married/stable union	1486 (62.0)	1456 (61.9)	30 (68.2)	Ref	Ref	Ref
Single, widow	829 (34.6)	816 (34.7)	13 (29.6)	0.78	0.41	1.48
Divorced	80 (3.3)	79 (3.4)	1 (2.3)	0.62	0.09	4.48
Educational level						
Low (< 8 years)	266 (11.1)	264 (11.2)	2 (4.6)	0.34	0.08	1.39
Intermediate (8–12 years)	1515 (63.2)	1481 (63.1)	34 (77.3)	Ref	Ref	Ref
High (> 12 years)	609 (25.4)	601 (25.6)	8 (18.2)	0.58	0.27	1.26
Occupation						
Employee	1019 (42.5)	999 (42.6)	20 (45.4)	Ref	Ref	Ref
Housewife	301 (12.6)	295 (12.6)	6 (13.6)	10.16	0.41	25.06
In education	985 (41.1)	968 (41.3)	17 (38.6)	0.88	0.46	16.69
Unemployed	82 (3.4)	81 (3.5)	1 (2.3)	0.62	0.08	45.72
Remuneration	1121 (46.8)	1102 (46.9)	19 (43.2)	1.16	0.64	2.09
Physical activity	979 (40.8)	956 (40.6)	23 (52.3)	1.58	0.88	2.85
Religious practice	1698 (70.8)	1668 (70.9)	30 (68.2)	0.88	0.47	1.65
Smoking	474 (19.8)	465 (19.8)	9 (20.4)	1.04	0.50	2.16
Alcohol use disorder	1358 (56.6)	1327 (56.4)	31 (70.4)	1.82	0.96	3.47
Illicit drug use	131 (5.5)	126 (5.4)	5 (11.4)	2.22	0.89	5.53
Violence victim	565 (23.6)	541 (23.0)	24 (54.6)	3.891	2.17	6.99
Abdominal surgery	668 (27.9)	649 (27.6)	19 (43.2)	1.96	1.09	3.54
Contraceptive use	620 (25.9)	610 (25.9)	10 (22.7)	0.84	0.42	1.70
Abnormal menstruation	431 (18.0)	418 (17.8)	13 (29.6)	1.91	1.01	3.62
Previous sexual intercourse	1909 (79.6)	1870 (79.5)	39 (88.6)	1.99	0.79	5.03
Parity						
0	1239 (51.7)	1221 (51.9)	18 (40.9)	Ref	Ref	Ref
1–2	747 (31.2)	730 (31.0)	17 (38.6)	1.57	0.81	3.02
3 +	411 (17.2)	402 (17.1)	9 (20.4)	1.51	0.68	3.33
Dyspareunia	210 (8.8)	199 (15.7)	11 (36.7)	2.99	1.45	6.20
Cardiovascular disease	142 (5.9)	139 (5.9)	3 (6.8)	1.16	0.36	3.71
Diabetes	33 (1.4)	32 (1.4)	1 (2.3)	1.67	0.24	11.74
Respiratory disease	176 (7.3)	170 (7.2)	6 (13.6)	1.99	0.85	4.65
Cancer	17 (0.7)	17 (0.7)	0 (0.0)	–	–	–
Psychiatric disorder	77 (3.2)	74 (3.1)	3 (6.8)	2.20	0.70	6.96
Migraine/chronic headache	552 (23.0)	538 (22.9)	14 (31.8)	1.56	0.83	2.92
Lombalgia	382 (15.9)	370 (15.7)	12 (27.3)	1.98	1.03	3.81
Irritative urinary symptoms	373 (15.6)	359 (15.3)	14 (31.8)	2.53	1.36	4.73
Constipation	482 (20.1)	479 (20.4)	3 (6.8)	0.29	0.09	0.93
Distension	937 (39.1)	910 (38.8)	27 (61.4)	2.46	1.35	4.49
IBS criteria	283 (11.8)	257 (10.9)	26 (59.1)	10.79	5.99	19.43
Bristol scale						
Types 1–2	675 (28.2)	662 (28.2)	13 (29.6)	1.10	0.57	2.10
Types 3–4–5	1650 (68.8)	1621 (68.9)	29 (65.9)	Ref	Ref	Ref
Types 6–7	71 (3.0)	69 (2.9)	2 (4.6)	1.60	0.39	6.58
Sleep disturbance	654 (27.3)	636 (27.0)	18 (40.9)	1.84	1.02	3.34
SRQ > = 8	425 (17.7)	404 (17.2)	21 (47.7)	4.24	2.37	7.58

*CI*confidence interval; *PR* prevalence ratio; *IBS* irritable bowel syndrome; *SRQ* self report questionnaire (used for screening mental disorders)

Women with primary dysmenorrhoea experienced more intense pain than women with non-cyclical pelvic pain (74.3 ± 22.6 mm versus 60.3 ± 19.7 mm, respectively, mean difference = 14.0 mm, 95% confidence interval 10.0–17.9, *p*-value < 0.001). The difference between catastrophising scores was not significant between women with primary dysmenorrhoea and women with non-cyclical CPP (12.8 ± 14.1 versus 11.1 ± 12.0, respectively, mean difference = 1.7, 95% confidence interval − 0.7 to 4.1, *p*-value = 0.166).

Women with primary dysmenorrhoea and concomitant non-cyclical pelvic pain (n = 44/2,397, 1.8%) had a higher average mean pain intensity (80.9 ± 17.8 mm) than women with non-cyclical pelvic pain alone (mean difference = 20.6 mm, 95% confidence interval 14.3–26.9, *p*-value < 0.001), but there was no significant difference in pain score compared to the scores of women with primary dysmenorrhoea alone (mean difference = 6.6, 95% confidence interval − 0.5 to 13.8, *p*-value = 0.068). The catastrophising score in women with primary dysmenorrhoea and non-cyclical pelvic pain was 16.9 ± 13.8, which was higher than that of women with non-cyclical pelvic pain alone (mean difference = 5.8, 95% confidence interval 1.8–9.7, *p* = 0.005), but not statistically different from those of women with primary dysmenorrhoea alone (mean difference = 4.06, 95% confidence interval − 0.5 to 8.6, *p* = 0.082). Additionally, there was a weak correlation between pain intensity and the catastrophising score in women with both primary dysmenorrhoea and non-cyclical pelvic pain (r = 0.07, *p* = 0.005).

## Discussion

We identified a prevalence of 9.8% for non-cyclical CPP and 8.9% for primary dysmenorrhoea in women of reproductive age in Ecuador. The main variables associated with CPP were irritative urinary symptoms, primary dysmenorrhoea, and mental disorders. The main variables associated with primary dysmenorrhea were smoking, IBS, sleep disturbance, dyspareunia, and mental disorders. To the best of our knowledge, this is the first study reporting the prevalence of these conditions and associated factors in Ecuador. Reiterating the purpose of this study, we believe that the integration of this information in the international literature can contribute to highlight the importance of CPP within the context of women's health worldwide, and help to map common association factors that may be addressed through global health policies. The observed prevalence of CPP in Ecuador was similar to that identified in other Latin American countries. In Brazil, the prevalence of CPP varied between 10% in affluent neighbourhoods [11] and 20% in disadvantaged regions [49]. In Mexico, the prevalence rates of CPP and dysmenorrhoea were 6% and 40%, respectively [50].

Our study showed that both CPP and dysmenorrhoea were independently associated with higher SRQ-20 scores. We consider this an important point; however, there is a limitation due to the absence of a formal validation of the instrument in this population. Several prospective studies have attempted to understand the causal relationship between mood and psychiatric disorders and pain and vice-versa. Despite the lack of conclusive evidence, particularly with regard to primary dysmenorrhoea [51, 52], the literature suggests that chronic pain may be a greater risk factor for the development of negative psychological symptoms than vice-versa [53]. In any case, the mutual occurrence of these conditions may be, at least in part, due to common neurobiological vulnerabilities and signify an affective relationship or emotional vulnerability to chronic pain [54]. Some authors have also correlated the symptoms of anxiety and depression with the catastrophising process. However, although a correlation is present, they are different constructs [55]. Catastrophising is considered an attitude of distress or belief in response to perceived pain, as opposed to a coping mechanism [56]. Moreover, a significant aspect of this relationship is that women with high catastrophising scores have worse rates of symptom relief in response to different treatment strategies [57, 58].

We also found that primary dysmenorrhoea was a potential risk factor of non-cyclical pelvic pain. This finding is in agreement with that of a prospective study [59] and a recent meta-analysis of population-based studies [60]. There is also some evidence of an association between structural and functional brain alterations [61] and abnormal reward neural system connectivity in patients with primary dysmenorrhoea [62]. These changes may be associated with the abnormal empathy observed in women with primary dysmenorrhoea, regardless of their phase of the menstrual cycle [63, 64]. Along with catastrophising, these neurological aspects can significantly affect the experience and intensity of pain that is perceived by women with primary dysmenorrhoea [65].

In addition, long-standing primary dysmenorrhoea can induce adaptive neuroplasticity with consequent functional reorganisation of the central nervous system neural networks which can result in central sensitisation [66]. This functional remodelling can have implications for pain modulation [67], potentially leading to lower pain thresholds or greater reactivity to painful stimuli [68]. This reduced neuroplasticity may explain the high frequency and independent association between primary dysmenorrhoea and IBS observed in our study [69]. Another interesting finding was the independent association between CPP and irritative urinary symptoms. However, contrary to our expectations, irritative urinary symptoms were not associated with primary dysmenorrhoea. Recent studies have reported persistent autonomic dysfunction and bladder sensitivity in primary dysmenorrhoea [70]. Our study may not have been sensitive to detect this, as we did not perform any type of provocative testing, nor did we standardise the phase of the menstrual cycle at the time of administering the questionnaire, as has been used in study designs that repeatedly observe this association [71, 72].

Further, less than a quarter of the women with primary dysmenorrhoea in this study were using hormonal contraceptives, which can effectively control this condition [73]. This may reflect that socioeconomic inequalities still exist in women’s access to healthcare in Ecuador, even after the reformation in the Ecuadorian health system [74]. Although it is plausible to state that primary dysmenorrhoea is a relevant risk factor of non-cyclical CPP, only longitudinal studies can confirm a causal association between these conditions.

Another relevant observation in our study was the association between smoking and primary dysmenorrhoea, although we cannot draw conclusions about the nature of this temporal relationship. A recent meta-analysis of observational studies showed that smokers were 1.45 times more likely to develop dysmenorrhoea than non-smokers [75]. Furthermore, it has been observed that there is a strong dose–response association between tobacco exposure and the intensity of dysmenorrhoea [76]. On the other hand, there seems to be a complex relationship between smoking, psychological symptoms, and primary dysmenorrhoea. Some studies have even counterintuitively observed that symptoms of anxiety and depression may have a higher impact on dysmenorrhoea in women who have never smoked [77]. A possible explanation for this may be that the substances found in cigarettes can antagonise the synthesis of prostaglandins, which may be associated with the genesis of dysmenorrhoea. Nevertheless, the bidirectional relationship between smoking, anxiety, and depression may have obscured and complicated the interpretation of these findings [78]. Regarding primary dysmenorrhoea, our cross-sectional study design does not allow us to assert a temporal association between smoking and the development of pelvic pain. Despite this, the information is relevant because it shows the need for a policy to reduce the consumption and frequency of cigarette use among women with pain which is approximately 30%, and is well above the rates observed in the general population of women of reproductive age in Ecuador [79].

The association between primary dysmenorrhoea and sleep disturbance is also relevant. Recent publications have highlighted the potential negative impact of primary dysmenorrhoea on sleep disturbance, but only few studies have reported on this relationship [80]. A recent review showed that patients with chronic pain and sleep disturbances are more likely to experience anxiety, depression, catastrophising, and suicidal ideation [81]. This relationship is believed to be intrinsically linked to the presence of central sensitisation and dysfunction of the dopaminergic, serotonergic, and opioidergic systems [82]. Moreover, even in healthy individuals, sleep deprivation can trigger an increase in pain sensitivity, impairment in conditioned pain modulation, and facilitation of the temporal summation process [83]. This is further evidence that neuroplasty is associated with chronic pain processes and that primary dysmenorrhoea is a potential event which can induce this process, as brain changes can occur early and rapidly in these women [84, 85].

### Implications for practice

Considering our study as a whole, primary dysmenorrhoea, in particular, draws our attention for its significant association with non-cyclical CPP, higher intensity of symptoms, and other elements suggestive of neuroplasty and central sensitisation. We believe that this may be an initial marker for the risk of pain progression to chronicity. Severely intermittent painful menstrual episodes can trigger a process of hyperalgesic priming, which in turn can lead to neuroplastic change in nociceptors and consequently, pain chronicity (Jarrel, Arendt-Nielsen AJOG 2016). Despite these negative aspects, our study shows that there is a window of opportunity to combat this condition, as only a quarter of women with the disease use contraceptives, which can relieve early symptoms and potentially prevent the progression of the condition or future deleterious associations. We believe that our population-based study provides evidence for the urgent need to determine whether early treatment of primary dysmenorrhoea is effective in reducing the intensity of associated symptoms, thereby reducing the risk of developing CPP. Finally, worldwide population studies are essential, not only to characterise the conditions, but also to alert the scientific and political community about the negative repercussions of these taboo subjects for women and society. This will allow the implementation of effective public health policies to understand and mitigate the problems associated with CPP and primary dysmenorrhoea.

## Conclusions

Our study shows that the prevalence of CPP and primary dysmenorrhoea in this population is high, and the latter should be considered as a risk factor for the former. Furthermore, there is an independent association with symptoms that can be interpreted as signs of central sensitisation, neuroplasty (irritative urinary symptoms, IBS, sleep disturbance, and mental disorders) and potentially aggravating behaviours, particularly smoking (Additional file 1).

## Supplementary Information

**Additional file 1**. Information from the questionnaire used for the interviews.

## Abbreviations

CPP: Chronic pelvic pain
IBS: Irritable bowel syndrome
SRQ-20: Self-reporting questionnaire 20
VAS: Visual analogue scale
